# The Effect of Social Communication on Life Satisfaction among the Rural Elderly: A Moderated Mediation Model

**DOI:** 10.3390/ijerph16203791

**Published:** 2019-10-09

**Authors:** Yashuo Chen, Chunjiang Yang, Shangjun Feng

**Affiliations:** 1 College of Business, Yantai Nanshan University, Yantai 265713, China; chenyashuo@stumail.ysu.edu.cn; 2 School of Economics and Management, Yanshan University, Qinhuangdao 066004, China; shangjunfeng@stumail.ysu.edu.cn

**Keywords:** rural elderly, life satisfaction, social communication, psychological well-being, emotional support

## Abstract

Life satisfaction of the rural elderly has increasingly become an important issue for society. Based on the social support theory and Cha Xu Ge Ju (pattern of difference sequence), this study investigates the underlying mechanisms and boundary conditions that explain the relationship between social communication and life satisfaction among the rural elderly. Specifically, it explores the mediating role of psychological well-being in the relationship between social communication and life satisfaction. In addition, it examines whether emotional support moderates the effect of social communication on psychological well-being. Data from 658 rural elderly in China were analyzed using structural equation modeling. Results show that psychological well-being mediates the relationship between social communication and life satisfaction. Additionally, the relationship between social communication on psychological well-being was negatively moderated by emotional support. Finally, implications for management theory and practice are discussed.

## 1. Introduction

The life satisfaction of the rural elderly has long been a key concern of government and scholars, especially in China. On the one hand, China has experienced a population transition from “high fertility and high mortality” to “low fertility and low mortality”. The aging of the population is now an irreversible trend, as fertility rates fall and life expectancies rise. By the end of 2018, China had 249.49 million people aged 60 or above, accounting for 17.9 percent of the total population [1]. On the other hand, as a result of the rapid economic growth for the four decades since the reform and opening up in 1978, China has been experiencing rapid urbanization created by the history’s largest flow of rural–urban migration in the world. During the process, the elderly can only continue to stay in rural areas because of economic pressures and traditional life concepts [2]. They have become the main body of rural residents. However, the current rural social pension guarantee system has not been generally established in China, and the traditional family support is still the main mode of elderly care. Rural youth from rural areas to cities leads to the spatial separation of the subject and object in the elderly life care relationship [3]. The rural elderly are facing more uncertainties in economic support, living care, spiritual support, and other aspects. Focusing on rural elderly’s life satisfaction has important theoretical and practical implications. In recent years, the Chinese government has worked hard to improve the quality of life for the elderly in rural areas. Specifically, the numbers of old-age care institutions are increasing to improve the dilemma of the rural elderly. The new rural cooperative medical system played an active role in meeting the rural elderly’s needs on health care, alleviating their difficulties in affording the medical costs and seeing doctors. In addition, all rural elderly over 60 years old can enjoy about 80 yuan of daily living allowance from the national finance every month.

In academia, scholars have long tried to determine predictors of life satisfaction of the rural elderly. Prior studies provide empirical evidence of the positive effects of social support, rural pension programs, loneliness, and others on the life satisfaction of rural elderly [4,5,6]. However, further research is needed regarding the antecedents, specific mechanisms by which these effects occur and the boundary conditions under which the antecedents influence the life satisfaction of the rural elderly. Therefore, this research aims to provide new insights into the problem of life satisfaction of the rural elderly in China from the perspectives of what, why, and under what circumstances. With the acceleration of China’s urbanization process, a large number of young and middle-aged rural labor force migrate to cities in order to obtain a higher living standard. Restricted by the incomes and living conditions, they can only leave their parents in the countryside, which leads to the emergence of rural empty-nesters. Due to the lack of fixed salary and single economic sources, rural elderly people need to rely more on their children for economic support. However, even more importantly, those rural elderly lack care and social support from their children, and one of the biggest problems they face is loneliness. Given the rather bleak picture just described, the life satisfaction of rural elderly has serious problems. Thus, the pursuit of happiness and the achievement of the ‘good life’ for the rural elderly may be an important concern for researchers. In response to these calls for further research, first, the study investigated the effect of social communication on life satisfaction. Society is viewed as clusters of people joined by a variety of links. Social communication describes the patterns of social relationships between people [7]. Lindsay found that social communication can reduce individuals’ loneliness and negative emotions and make people feel warm [8]. Second, this study draws on social support theory to explore the mediating role played by the psychological relationship between social communication and life satisfaction, in terms of the rural elderly’s psychological well-being. Psychological well-being is related to the individual’s optimal psychological functioning and experience [9]. Third, this study investigates the boundary conditions that may moderate the relationship between social communication and psychological well-being. Emotional support (e.g., making someone feel valued, loved, and cared for) affects support recipients. However, much less is known about emotional support in daily life and the effects of support provision on recipients. Here, we address these gaps in knowledge by examining whether emotional support moderates the relationship between social communication and psychological well-being [10]. This study provides several contributions to the academic literature and to managerial practice and we elaborate on these and other contributions in the discussion section.

## 2. Theory Framework and Hypotheses

### 2.1. The Mediating Role of Psychological Well-Being

#### 2.1.1. Social Communication and Psychological Well-Being

Social communication is a supportive interpersonal communication system building among individuals. Social communication is a process in which people establish connections with others through mutual contacts, actions, and influences. The social support theory posits that the stronger the social support network a person has, the better able he or she is to cope with all kinds of life challenges [11]. According to social support theory, social communication would improve an individual’s psychological well-being [12]. Wide and close social communication can help individuals build a high-quality social network that provides intangible and tangible resources to the rural elderly. Therefore, the rural elderly will get more external information and help, tend to interpret events from a positive perspective, and reduce the psychological pressure caused by negative events. Shimada has found that positive social communication can improve the elderly’s well-being and relieve negative emotions such as loneliness, helplessness, and depression [13]. In fact, in the process of rapid urbanization and industrialization, more and more of the rural youth labor force rush to the city for high economic income, while more and more elderly people are forced to stay in the countryside. As the modernization and vulnerability hypothesis [14,15] points out, the social status of the elderly has largely weakened. The rural elderly generally lack emotional communication and care from the family [16]. In addition, the rural elderly are facing low income, low education level, terrible medical care and welfare, and information occlusion. Accordingly, interpersonal communication between the elderly and their neighbors and relatives has become the main channel to meet the psychological and social needs of the rural elderly. On the one hand, social communication can alleviate the negative effects of negative events, because the network of interpersonal relationships not only provides the necessary material assistance in solving practical difficulties but also provides sympathy and comfort from the spiritual aspect, alleviating anxiety and loneliness. On the other hand, network relationships can meet the needs of being cared for, being respected and social belonging. Zeng and Gu [17] showed that social communication is one of the main influencing factors affecting the psychological well-being of the elderly. Thus, we propose:

**Hypothesis** **1.** *Social communication has a positive effect on psychological well-being.*

#### 2.1.2. Psychological Well-Being and Life Satisfaction

Psychological well-being concerns the full growth and self-realization of the individual through the resolution of existential life challenges. For instance, according to Ryff [18], psychological well-being comprises six dimensions: being independent and self-determining (autonomy), having a sense of control over the external world (environmental mastery), maintaining positive relationships with others (positive relations), finding meaning in one’s life (purpose in life), seeing oneself as growing and expanding (personal growth), and feeling good about oneself (self-acceptance) [19]. People evaluate the events in life according to the established standards, and then generate emotional experience and psychological activities, and thus make a comprehensive judgment on the quality of life. Ryff and Keyes [20] found that psychological well-being was positively related to life satisfaction. In addition, more and more evidence shows that the active and healthy spirit and mentality of the elderly improves their life satisfaction [21]. The fundamental reason is that the subjective evaluation of the quality of life is made by the elderly based on their psychological characteristics and mental state in a particular situation. Thus, we postulate:

**Hypothesis** **2.** *Psychological well-being has a positive effect on life satisfaction.*

#### 2.1.3. Social Communication, Psychological Well-Being, and Life Satisfaction

As we noted earlier, psychological well-being has six dimensions. In terms of positive relations, frequent social communication can improve the intimacy, harmony, and coordination of interactions with others and then promote the establishment of good relationships. Having a good interpersonal relationship not only provides the elderly with financial and material support but also provides them with a social platform for interpersonal communication and seeking a sense of belonging [22]. In other words, the material and spiritual needs of the rural elderly can be satisfied through positive relations, and thus improve their life satisfaction. In terms of self-acceptance, the individual’s self-acceptance is evaluated by his role, status, and experience through social communication. Social communication can improve the sense of belonging and self-acceptance and enhance their positive cognition of life [23]. In terms of purpose in life, based on the self-determination theory, individuals can achieve self-growth through social communication activities [24]. Obviously, the realization of life goals will undoubtedly improve the satisfaction of individuals with real-life conditions. In terms of environmental mastery, social communication improves the material and economic conditions, family atmosphere, and interpersonal relationships. The good environmental atmosphere and social network enhance the individual’s environmental control ability, reduce the risk brought by environmental uncertainty, and thus improve the individual’s self-determination and subjective evaluation of real life. In terms of autonomy, social communication enables individuals to gain more social support, and thus enhance their confidence [25]. Self-determination theory argues that individual autonomy is more likely to stimulate satisfaction [26]. Finally, in terms of personal growth, social communication helps the elderly to reduce the risk of mental illness, achieve psychological health, obtain information, accumulate new experiences, and thereby strengthen the positive evaluation of life satisfaction.

In addition, previous studies on social communication of rural residents have found that social communication improved individual mental health [27], generating more positive self-health evaluation [28]. It also helped to construct and expand social capital, promote the development of human nature [29], and social psychological merger [13]. Under the influence of these positive effects above-mentioned, the rural elderly maintain a healthy psychological state and a positive self-evaluation and then improve the subjective cognition of life satisfaction. As noted by Li, the elderly who actively engage in social communication build a closer and broader network of social relationships [30,31,32], which not only provide the elderly with material help and spiritual support/comfort in the face of difficulties, but also improve their happiness and help them to maintain/restore an optimistic attitude to evaluate the quality of life. Furthermore, integrating these arguments and Hypotheses 1 and 2, thus, we propose the following hypothesis:

**Hypothesis** **3.** *Psychological well-being mediates the relationship between social communication and life satisfaction.*

### 2.2. The Moderating Effect of Emotional Support

**Figure 1:**
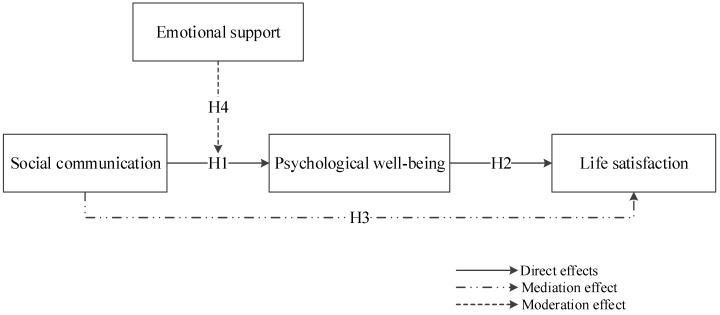
Theoretical model.

Intergenerational support is a variety of help provided for the elderly by their children or grandchildren in the family, including financial support, life care, and emotional support [33]. In the study, we mainly focused on emotional support which is the emotional comfort that the children provide to the elderly by talking, sharing, and caring. It is an important indicator to measure the harmonious relationship between the elderly and their children [34]. In the Chinese context, influenced by traditional Chinese Confucianism, filial piety is one of the core contents in Chinese traditional culture. That is, having the duty of filial piety is important for children, as well as the elderly expecting filial piety from the children. The primary tenet of Cha Xu Ge Ju (pattern of difference sequence) of Mr. Fei [35] is that individuals are self-centered, and they construct a social network circle of diminishing relationships according to relationships from close to shallow with the surrounding society. This kind of social relationship is centered on the blood standard and family standard, which expands from the family to the outsiders, among which the blood relationship is the most intimate. Specifically, for the rural elderly, their social communication circles, including spouses, children, relatives, neighbors, and acquaintances and so on. Those people would provide psychological well-being for the elderly. If children provide adequate emotional support for the elderly, it will undoubtedly reduce the need for the elderly to rely on external social communication to obtain psychological well-being. Wang argued that the elderly had the most frequent communication with their children [36], and living together with their children is also the hope of the rural elderly in China. However, in fact, young laborers in rural areas are mostly working in urban areas, while the elderly are staying in rural areas [37]. As a result, the rural elderly generally lack emotional support compared with the urban elderly, because family support is difficult to satisfy. The rural elderly tend to get emotional support and psychological needs from relatives, neighbors, and other social relationships. In other words, the elderly will acquire psychological needs from both “family” and “outsiders”, when it is difficult to be satisfied from the “family”, they will retreat to “outsiders” to obtain psychological well-being. Thus, the family’s emotional support may affect the relationship between social communication and the psychological well-being of the rural elderly. We proposed the Hypothesis 4. Moreover, the overall theoretical model was depicted in Figure 1.

**Hypothesis** **4.** *Emotional support moderates the relationship between social communication and psychological well-being. When emotional support is low, the positive relationship between social communication and psychological well-being is stronger, and vice versa.*

## 3. Method

### 3.1. Participants and Procedures

We invited residents from the townships of four prefecture-level cities (Qinhuangdao, Shijiazhuang, Chengde, and Baoding) in Hebei Province for field investigations. Limited by social resources, the selection of samples was random as much as possible in the principle of convenience to minimize sample bias. The researchers selected 4–6 natural villages in each of the above four places and selected 20–30 residents from each village to participate in the survey. In the current study, a total of 707 questionnaires were distributed and 658 valid questionnaires were obtained after excluding the invalid responses, with an effective response rate of 93.07%. During the investigation, considering that it was difficult for some elderly to complete the survey due to the limitations of their educational level and health status, we took a “one-on-one” form for those people to help them finish the questionnaire. 

In effective subjects, 369 were males (56.1%). Regarding age, 46.5% were aged between 60 and 69, 40.4% were aged between 70 and 79, 13.1% were aged above 80. In terms of marriage, only a few elderly people were unmarried or divorced (4.2%), among the married participants, 205 were widowed (31.2%). Regarding the living situations, 52.2% lived with their spouses, 23.9% lived with their children, 5% lived with their grandchildren, and 18.9% lived alone.

### 3.2. Measures

To ensure the reliability and validity of the measurement of each variable, we selected a mature scale which is suitable for domestic cultural background and widely used in the present study, SPSS18.0 and MPLUS7 statistical software packages were used to analyze the data.

Social communication. Based on the practices of Wei and Jia [28], social communication was measured using two items: “Do you often go to your neighbor’s house to chat or go out to chat with people in the village?”; 1 = (not often), 5 = (often). An example item of the contact objects is: “Who usually chat with you?”. The items are given 1–4 points from “children” to “friends”. The above two sums are social communication scores, the higher the score, the higher the degree of social communication.

Emotional support. Emotional support was measured by the three-item scale developed by Wang [38]. A sample item is “Generally speaking, you feel that you get along well with your children.” These items were measured using a five-point Likert scale, ranging from 1 (disagree) to 5 (agree). The Cronbach’s alpha coefficient of the scale is 0.863 (*χ*^2^/*df* = 1.462, CFI (Comparative Fit Index) = 0.998, TLI (Tacker–Lewis index) = 0.996, RMSEA (root mean square error of approximation) = 0.037).

Psychological well-being. We selected nine items from the CES-D Psychological Depression Scale [39], including “I often feel lonely”, “I always feel old and useless”. All items could be rated on a 5-point scale ranging from 1 (agree) to 5 (disagree). The Cronbach’s alpha coefficient of the scale is 0.932 (*χ*^2^*/df* = 9.669, CFI = 0.888, TLI = 0.856, RMSEA = 0.163).

Life satisfaction. Life satisfaction was measured with five items developed by Dinner et al. [40]. A sample item is “In most cases, my life is close to what I want to have”. All items could be rated on a 5-point scale ranging from 1 (agree) to 5 (disagree). The Cronbach’s alpha coefficient of the scale is 0.837 (*χ*^2^*/df* = 12.27, CFI = 0.952, TLI = 0.919, RMSEA = 0.148).

Gender was dummy coded as 1 = female, 0 = male. Spouse situation was dummy coded as 1 = have a spouse, 2 = mateless. The housing situation was dummy coded as 1 = yes (have your own housing), 2 = no (Don’t have your own housing). All items were shown in Appendix A in detail.

## 4. Data Analysis

### 4.1. Common Method Variance

Considering the possibility of common method bias, several recommendations in the literature were adopted including the anonymity of research objects used in the survey [41]. In addition, we set the four latent variables of social communication, emotional support, psychological well-being, and life satisfaction as a common factor. The results of confirmatory factor analysis showed that the single factor model was difficult to fit (*χ*^2^/*df* = 16.13, RMSEA = 0.142, CFI = 0.722, TLI = 0.691), indicating that the single factor was difficult to explain most of the variation, and there was no serious common methodological bias in the current study.

### 4.2. Confirmatory Factor Analyses

Confirmatory factor analyses were conducted to test whether the constructs among this study had good convergent validity and discriminate validity. Results in Table 1 show that the square root of the average variance extracted (AVE) for every reflective component above 0.7 offers satisfying evidence of discriminant validity because they were larger than all the bivariate correlation coefficients among the components. We constructed seven alternative models in addition to the theoretical model. M_1_, M_2_, and M_3_ were three-factor models, which were social communication combined with emotional support, social communication combined with psychological well-being, and social communication combined with life satisfaction, respectively. M_4_, M_5_, M_6_, and M_7_ were two-factor models, In M_4_, social communication, psychological well-being, and emotional support were combined into one factor. In M_5_, social communication, emotional support, and life satisfaction were combined into one factor. In M_6_, social communication, psychological well-being, and life satisfaction were combined into one factor. In M_7_, emotional support, psychological well-being, and life satisfaction were combined into one factor. Results in Table 2 show that the four-factor model has the best model fit when compared with other alternative models (χ^2^/df = 2.673, RMSEA = 0.071, CFI = 0.949, TLI = 0.940). For example, the two-factor model (M_6_) reveals a poor fit (χ^2^/df = 10.034, RMSEA = 0.133, CFI = 0.754, TLI = 0.715).

### 4.3. Hypothesis Testing

According to Muller’s three-step test of the moderated mediation model [42], we constructed the following regression models:

(1) Regression of life satisfaction to social communication, emotional support, and the interaction term of emotional support and social communication, as shown in Formula (1):

(1) $$\begin{array}{l} {Y\;=\;c}_{0}{+\;c}_{1}{X\;+\;c}_{2}{U\;+\;c}_{3}{\mathrm{UX}\;+\;u}_{1}. \end{array}$$

Y is life satisfaction, X is social communication, and U is emotional support. c_0_ is a constant term, c_1_, c_2_, and c_3_ are regression coefficients for each variable, and u_1_ is a regression residual term.

(2) Regression of psychological well-being on social communication, emotional support, and interaction term of emotional support and social communication, as shown in Formula (2):

(2) $${W\;=\;a}_{0}{\;+\;a}_{1}{X\;+\;a}_{2}{U\;+\;a}_{3}{\mathrm{UX}\;+\;u}_{2}.$$

W is psychological well-being, a_0_ is a constant term, a_1_, a_2_, a_3_ are regression coefficients for each variable, and u_2_ is a regression residual term.

(3) Regression of life satisfaction on social communication, emotional support, psychological well-being, and interaction term of emotional support and social communication, as shown in Formula (3):

(3) $$Y\;=\;{c^{\prime }}_{0}\;+\;{c^{\prime }}_{1}X\;+\;{c^{\prime }}_{2}U\;+\;{c^{\prime }}_{3}{\mathrm{UX}\;+\;b}_{1}{W\;+\;u}_{3}.$$

c′_0_ is a constant term, c′_1_, c′_2_, c′_3_, and b_1_ are regression coefficients for each variable, and u_2_ is a regression residual term.

According to the above formulas, we first examined the moderating effect of emotional support. We use the pairing method to calculate interaction terms (UX) and established regression model Ma of social communication (X), emotional support (U), and the interaction term (UX) on life satisfaction (Y). The results are shown in Table 3. Social communication had a positive effect on life satisfaction (c_1_ = 0.394, t = 6.880, *p* < 0.001), interaction term had a negative effect on life satisfaction (c_3_ = −0.300, t = −2.325, *p* < 0.05). Thus, Hypothesis 4 was supported.

We also constructed regression model Mb of social communication (X), emotional support (U), and interaction term (UX) on psychological well-being (W) and regression model Mc of social communication (X), emotional support (U), psychological well-being (W), and interaction term (UX) on life satisfaction (Y). As the results show in Table 3, in model Mb, social communication had a positive effect on psychological welfare (a_1_ = 0.588, t = 16.104, *p* < 0.05). Thus, Hypothesis 1 was supported. In model Mc, social communication had a significant influence on life satisfaction (c’_1_ = 0.242, t = 2.291, *p* < 0.05), and interaction term (UX) had a significant negative influence on life satisfaction (a_3_ = −0.278, t = −2.024, *p* < 0.05), but the influence of psychological well-being on life satisfaction was not significant (b_1_ = 0.140, t = 1.206, *p* > 0.05). In other words, a_3_b_1_ was not significant. Thus, Hypothesis 2 was not supported.

To further test the Hypothesis 3, bootstrap analysis procedures suggested by Ye [43] were used. We calculated the confidence interval for a_1_b_1_ and a_3_b_1_, if the confidence interval excluded 0, it indicates that the mediation and the moderated mediation effect exist [42]. The bias-corrected bootstrapped 95% confidence interval for a_1_b_1_ excluded zero (0.061, 0.157), indicating that the indirect effect of social communication on life satisfaction via psychological well-being was significant, thus, Hypothesis 3 was supported. Bias-corrected bootstrapped 95% confidence interval for a_3_b_1_ excluded zero (0.052, 0.103), indicating that the moderated mediation effect was significant and the moderated mediation effect was supported. The results are shown in Table 4.

As the results show in Table 3, the total effect of the interaction of social communication and emotional support on the life satisfaction part was −0.300, in which the direct effect c’_3_ was −0.278, and the indirect effect was c_3_ − c’_3_ = −0.022. The ratio of the indirect effect in the total effect was −0.022/−0.300 = 0.07. When the independent variable of psychological well-being was entered into the regression, the direct effect of interaction term on life satisfaction remained significant (c’_3_ = −0.278, t = −2.024, *p* < 0.05), indicating that psychological well-being partially mediated the relationship between social communication and life satisfaction, and Hypothesis 3 was further supported.

**Figure 2:**
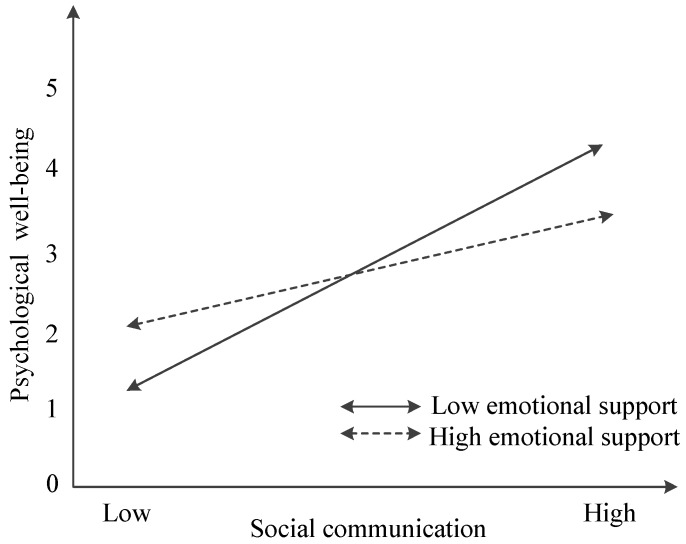
The moderating role of emotional support in the relationship between social communication and psychological well-being.

Figure 2 graphically represents the moderating effect of emotional support between social communication and psychological well-being. To facilitate interpretation, we plotted the interaction (Figure 2) and calculated the simple slopes (−1 SD; +1 SD). As predicted, the relationship between social communication and psychological well-being was stronger at lower levels of emotional support (β_low_ = 0.578, *p* < 0.001), rather than at higher levels of emotional support (β_high_ = 0.409, *p* < 0.001).

## 5. Discussion

Based on the social support theory and Cha Xu Ge Ju, we examined models linking social communication to life satisfaction that included psychological well-being as a mediator and emotional support as a moderator. Our results showed that social communication was associated with psychological well-being. Psychological well-being as a linking mechanism, providing an explanation as to the process by which social communication improves life satisfaction. Furthermore, our results indicated a negative interaction between social communication and emotional support in psychological well-being. We found that when emotional support is low, the relationship between social communication and life satisfaction is strong, but this relationship will be attenuated when emotional support is high. These results offer glimpses into the mechanisms by which social communication manifests itself in life satisfaction and the boundary conditions surrounding its effectiveness.

This research contributes to the literature and practice in several ways. We conclude that the life satisfaction of the rural elderly in China is an important and serious social problem. Specifically, we focus on the antecedents, mechanisms, and boundary conditions of the rural elder’s life satisfaction. Because of this, we constructed a connection between social communication and life satisfaction and revealed the psychological process of high-level social communication promoting positive evaluation of the quality of life for the rural elderly. Life satisfaction is a comprehensive psychological indicator for a subjective evaluation of the individual’s quality of life, which is also the result of improving living conditions and pursuing individual and family needs. From this perspective, we regard the rural elderly’s life satisfaction as an important component of life improvement and social development. In particular, the life satisfaction of the rural elderly is important for building a harmonious society [40]. People’s individual behaviors and social behaviors are based on subjective perceptions of objective life. Therefore, on the basis of previous research, we regard social communication as a predictor of life satisfaction and expound how social communication can improve an individual’s psychological well-being and then affect their life satisfaction.

Second, in response to calls for more research into the different influence processes involved in social communication and life satisfaction, this study explores the underlying mechanisms that link social communication and life satisfaction among the rural elderly. We proposed that psychological well-being mediated the relationship between social communication and life satisfaction. Those rural elderly who actively participate in social activities frequently would receive support from others. Therefore, they can build a high-quality social network which can effectively alleviate the pressure of negative events, gain more sense of belonging and caring, and have positive psychological health and life evaluation. 

Third, few studies have investigated the potential boundary conditions that qualify the relationship between social communication and psychological well-being. Although previous research has underlined the importance of emotional support, to the best of our knowledge researchers have not yet explored the moderating role of emotional support on the relationship between social communication and psychological well-being. Based on Cha Xu Ge Ju, we proposed and tested the moderating effect of emotional support in the relationship between social communication and psychological well-being. The results show that emotional support significantly moderates the relationship between social communication and psychological well-being, when the rural elderly had more emotional support, the effect of social communication on psychological well-being was relatively weak. In other words, emotional support negatively moderated the relationship between social communication and psychological well-being. Emotional support, as an emotional reward from children, affects the sensitivity of the rural elderly to external social communication, thus those rural elderly who are less likely to receive emotional support from their children will rely more on social communication with external members. Because they will get support, identity, belonging and love from the external interpersonal relationship, which will affect their psychological state, attitude, and behavior.

Fourth, we found that the impact of the interaction between social interaction and emotional support on life satisfaction is also mediated by psychological well-being. That is, the interaction between emotional support and social communication affects life satisfaction through the psychological state of the rural elderly. Family support and social support constitute the two main ways to meet the social needs of the rural elderly. Under the background of Confucian “filial piety” culture, the emotional support from the family are the most anticipated by the rural elderly. Because the family not only provides security and care for the elderly but also includes filial piety, status, and authority. In sum, the sense of belonging, security, and identity of the elderly depend jointly on the family and the society, and thereby influence their psychological state and life evaluation.

Finally, the present study has practical implications that highlight the role of social communication in improving the life satisfaction of the rural elderly. Social needs of the rural elderly such as being cared for, being recognized and belonging are satisfied through social communication, and then develop good psychological well-being. As a result, social communication creates the necessary psychological conditions for the elderly to positively evaluate the quality of life. A dilemma prevails among rural families. On the one hand, young and strong laborers need to support the elderly and their children. On the other hand, for employment and higher economic income, they have to leave their parents and children to work in cities. We believe that the dual economic structure is difficult to change in a short period of time. Based on the findings of this study, social communication provides a certain idea to alleviate the dilemma. We should give full play to the role of interpersonal communication, using the relationship network to provide objective support such as economic and information support for the elderly, as well as providing spiritual support such as mutual care. In addition, we can enhance the rural elderly’s subjective well-being and promote the harmonious development of society. 

## 6. Limitations and Future Directions

The present research has several limitations. First, although we examined the psychological mechanism (psychological well-being) in the relationship between social communication and life satisfaction, this variable does not fully reflect the entire psychological process of the effect of social communication on the life satisfaction of the rural elderly. For example, Li [44] proposed that social self-esteem plays a mediating role in social communication and life happiness. It would be worthwhile to explore other potential mediators from different theoretical perspectives. Second, limited by human, material, financial resources and other factors, the present study adopted the convenience sampling method to collect data rather than the random sampling. Due to the fact that the convenience sample may not be enough to represent the population of interest, the findings of this study may just, to some extent, apply to the population. Future research can use additional samples to investigate the validity and transportability of our findings. Considering the moderating role of emotional support, and China is a country that values family filial piety, our conclusions may have some contextual dependence, therefore, replicating these results in other countries/cultures or at different levels, will be important. Third, some covariates such as educational level, lifestyle, and medical history may confound our findings. Future studies could control those covariates or confounding factors in order to draw more precise conclusions.

## Figures and Tables

**Table 1:** Descriptive statistics and correlations for all measures (N = 658).

Variable	1	2	3	4	5	6	7	8
1. Gender	－	－	－	－	－	－	－	－
2. Age	−0.048	－	－	－	－	－	－	－
3. Spouse situation	−0.001	−0.025	－	－	－	－	－	－
4. Housing situation	0.009	0.030	0.020	－	－	－	－	－
5. Social communication	−0.004	−0.054	0.065	0.146 **	(0.807)	－	－	－
6. Emotional support	0.068	0.011	0.232 **	0.040	0.409 **	(0.807)	－	－
7. Life satisfaction	0.013	−0.010	0.145 **	−0.022	0.389 **	0.617 **	(0.794)	－
8. Psychological well-being	0.000	−0.016	0.084	0.070	0.599 **	0.537 **	0.526 *	(0.773)
**M**	0.5	71.12	1.16	1.83	4.89	3.69	2.99	3.48
**SD**	0.501	40.87	0.784	0.387	1.25	1.03	1.17	0.91
**Min**	0	60	1	1	1	1	1	1
**Max**	1	95	2	2	9	5	5	5

Note. * *p* < 0.05. ** *p* < 0.01. Square roots of AVE for each construct are reported on the diagonal.

**Table 2:** Comparison of measurement models.

Model	χ^2^/df	CFI	TLI	RMSEA
Baseline model	2.673	0.949	0.940	0.071
M_1_	5.659	0.874	0.853	0.095
M_2_	5.324	0.883	0.864	0.092
M_3_	6.981	0.838	0.811	0.108
M_4_	8.087	0.807	0.776	0.118
M_5_	8.828	0.787	0.753	0.124
M_6_	10.034	0.754	0.715	0.133
M_7_	8.393	0.799	0.767	0.120

**Table 3:** The results of the mediating effect and the moderating effect.

Model	Ma	Mb	Mc
Dependent variable	Y: life satisfaction	W: psychological well-being	Y: life satisfaction
Social communication(X)	0.394 (0.000)	0.588 (0.037)	0.242 (0.022)
Emotional support(U)	0.638 (0.000)	0.528 (0.047)	0.639 (0.000)
Social communication × emotional support (UX)	−0.300 (0.020)	−0.356 (0.000)	−0.278 (0.043)
Psychological well-being (W)	-	-	0.140 (0.228)

**Table 4:** Bootstrap analysis test.

Path	Indirect Effect	95% Confidence Interval	
		LL95%CI	UL95%CI
Social communication-psychological well-being-life satisfaction	0.110	0.061	0.157
Social communication × emotional support-psychological well-being-life satisfaction	0.075	0.052	0.103

Note. LL 95% CI = lower 95% level confidence interval; UL 95% CI = upper 95% level confidence interval.

## Appendix A. Partial Questionnaire

**Table A1:** A list of constructs and measured items.

Constructs	Items
Social communication	Do you often go to your neighbor’s house to chat or go out to chat with people in the village? 1 = never, 2 = seldom, 3 = sometimes 4 = often, 5 = everyday
	Who usually chat with you? 1 = children, 2 = relatives, 3 = neighbors, 4 = friends
Psychological well-being (1 = disagree; 5 = agree)	I feel good every day.
	I feel like every day is fine.
	I find life interesting.
	I often feel lonely.
	I always feel sad in my heart.
	I always feel old and useless.
	Now, I always feel like I have nothing to do.
	I always feel like I don’t want to eat, and it’s tasteless.
	I can’t sleep well (dream a lot/wake up early/insomnia).
Emotional support (1 = disagree; 5 = agree)	All things considered, you feel very close to your children.
	Generally speaking, you feel that you get along well with your children.
	When you talk to your child about your worries or difficulties, you think he/she is willing to listen to you pour out them.
Life satisfaction (1 = disagree; 5 = agree)	In most cases my life is close to what I want to have.
	My living conditions are very good
	I am satisfied with my life
	By far, I’ve got the most important thing I ever wanted in my life.
	If I had my life to live over, I would change almost nothing.
